# PFOS and PFOA in Humans: New Study Links Prenatal Exposure to Lower Birth Weight

**DOI:** 10.1289/ehp.115-a550a

**Published:** 2007-11

**Authors:** Kellyn Betts

Scientists have accumulated a wealth of evidence that perfluorooctane sulfonate (PFOS) and perfluorooctanoate (PFOA, also known as perfluorooctanoic acid) accumulate in the environment and humans. Animal studies have shown these compounds to cause a variety of health effects, including reduced birth size and infant mortality. Now researchers are presenting the first evidence to suggest that human exposure to the chemicals is linked to reduced birth weight **[*EHP* 115:1670–1676; Apelberg et al.]**.

PFOS, PFOA, and related polyfluoroalkyl compounds (PFCs) that can be transformed into these chemicals in the environment are used in a wide range of consumer applications, including oil and water repellents for fabric, apparel, and carpets, and paper coatings such as fast-food wrappers. The chemicals have been found in the blood of people throughout the world.

The new study shows that infants born with higher concentrations of PFOS and/or PFOA in their umbilical cord serum (a measure the researchers used as a marker of *in utero* exposure) had lower birth weights. The authors calculate the reduction as −69 g for PFOS and −104 g for PFOA. The study population included 293 infants born in Baltimore, Maryland, in 2004 and 2005. In earlier research, PFOA had been found in all these infants, and PFOS had been found in 99% of them. Infants with higher levels of PFOS and PFOA also had smaller head circumferences and lower scores on the ponderal index, a measure of body mass at birth. The study was not designed to allow the cohort to be followed in the future.

The results are consistent with those of toxicologic studies conducted with mice and rats that also have linked exposure to PFOS and PFOA with low birth weight—albeit at doses that resulted in much higher body burdens than those seen in the Baltimore infants. Both compounds have also been tied to developmental delays in animal studies. Previous studies in humans have correlated low birth weight with obesity, diabetes, and cardiovascular diseases later in life.

The researchers stress that the effect was small but statistically significant. They also note that the concentrations of PFOS, PFOA, and other PFCs in the infants’ blood were relatively low compared with levels tested in other studies.

The results were statistically adjusted to consider other potential sources of altered birth weight, such as maternal smoking, diabetes, and hypertension. The new study did not find a correlation between levels of the compounds and socioeconomic status, as had previous research. The researchers also found no evidence of an association between the infants’ exposure to the chemicals and their cholesterol or triglyceride levels, despite previous human and animal studies suggesting that these blood lipids are particularly sensitive to PFC exposure.

